# Association between Proton Pump Inhibitor Use and Risk of Incident Chronic Kidney Disease: Systematic Review and Meta-Analysis

**DOI:** 10.3390/biomedicines12071414

**Published:** 2024-06-25

**Authors:** Song Peng Ang, Jia Ee Chia, Carlos Valladares, Shreya Patel, Daniel Gewirtz, Jose Iglesias

**Affiliations:** 1 Department of Medicine, Rutgers Health Community Medical Center, Toms River, NJ 08755, USA; carlos.valladares@rwjbh.org (C.V.); daniel.gewirtz@rwjbh.org (D.G.); 2 Department of Internal Medicine, Texas Tech University Health Sciences Center, El Paso, TX 79905, USA; jchia@ttuhsc.edu; 3 Touro College of Osteopathic Medicine, New York, NY 10027, USA; spatel70@student.touro.edu; 4 Department of Medicine, Hackensack Meridian School of Medicine, Nutley, NJ 07110, USA

**Keywords:** CKD, ESRD, PPI, H2RA, pantoprazole, omeprazole

## Abstract

Introduction: Proton pump inhibitors (PPIs) are among the most commonly prescribed medications. Recently, PPI use has been linked to the development of chronic kidney disease (CKD) and cardiovascular events. Our study aimed to investigate the relationship between PPI use and the incidence of chronic kidney disease using a systematic review and meta-analysis. Methods: We performed a comprehensive literature search in PubMed, Embase, and Cochrane databases from their inception until March 2024 for relevant studies. We compared outcomes between patients using PPIs, those not using PPIs, and those using histamine-2 receptor antagonists (H2RAs). Endpoints were pooled using the DerSimonian-and-Laird random-effects model as the hazard ratio (HR) with 95% confidence intervals (CIs). Results: Our analysis included twelve studies with a total of 700,125 participants (286,488 on PPIs, 373,848 not on PPIs, and 39,789 on H2RAs), with follow-up periods ranging from three months to 14 years. The current meta-analysis revealed that PPI use is associated with a statistically significant increased risk of incident CKD (HR: 1.26, 95% CI: 1.16–1.38, *p* < 0.001) compared with non-users. Moreover, the risk of incident CKD is significantly higher in patients with PPI use compared to H2RA use (HR: 1.34, 95% CI: 1.13–1.59, *p* < 0.001). The results remained unchanged in terms of magnitude and direction after a leave-one-out analysis for both outcomes. Conclusions: Our multifaceted analysis showed that PPI use was associated with a higher incidence of CKD when compared to non-PPI use and H2RA use, respectively. These findings advocate for heightened vigilance and judicious use of long-term PPIs. Further large prospective longitudinal studies are warranted to validate these observations.

## 1. Introduction

Proton pump inhibitors (PPIs) are among the most commonly prescribed medications worldwide [1,2]. These agents are highly effective at inhibiting acid secretion and are generally well tolerated, with minimal short-term adverse effects [3]. However, the long-term use of PPIs should be limited to specific acid-related conditions, such as Zollinger–Ellison syndrome and Barrett’s esophagus [4,5] For most other scenarios, it is recommended to discontinue PPIs within 4 to 8 weeks of initiation. Despite these guidelines, studies indicate that PPIs are often overprescribed, particularly to patients managing multiple medications, and are sometimes prescribed for extended periods without a clear medical justification [6,7].

Over the past decade, there have been increasing concerns about the long-term adverse effects of PPIs [8]. Several studies have indicated a correlation between the use of PPIs and a heightened risk of cardiovascular events or mortality [9,10]. While these risks have garnered significant attention, the renal adverse effects of PPIs are less frequently discussed and might remain under-identified. Adverse renal outcomes frequently associated with proton pump inhibitor (PPI) use include hypomagnesemia, acute interstitial nephritis, and acute kidney injury [11]. There is a hypothesis suggesting that chronic usage of PPIs may precipitate the onset of chronic kidney disease (CKD), potentially through repeated episodes of acute kidney injury (AKI) [12].

Nevertheless, research findings related to this hypothesis are limited and inconsistent [13]. In light of the existing knowledge gap regarding the association between PPI use and CKD development, we conducted a systematic review and meta-analysis to explore the relationship between PPI use and the risk of developing CKD.

## 2. Methods

Our study was reported following the Preferred Reporting Items for Systematic Review and Meta-analysis (PRISMA) 2020 guidelines [14]. The study was registered in the International Prospective Register of Systematic Reviews (PROSPERO) (Identifier: CRD42024529654).

### 2.1. Search Strategy

A systematic literature search was performed in PubMed and Embase for articles from the journals’ inception until March 2024, using the following keywords: “proton pump inhibitors”, “dexlansoprazole”, “esomeprazole”, “lansoprazole”, “omeprazole”, “pantoprazole”, “rabeprazole”, “chronic kidney disease”, “end-stage renal disease”, and “renal failure”. The titles and abstracts were initially screened by two authors (SPA and JEC), who then proceeded to assess the full articles for their inclusion criteria. During the screening and selection phases, any disagreements were reconciled by a third author (JI).

### 2.2. Inclusion and Exclusion Criteria

The studies included in the analysis satisfied several criteria: (i) they involved participants aged 18 years or older; (ii) they documented the use of proton pump inhibitors (PPIs); (iii) they were two-arm studies, with the control groups consisting of either non-PPI users or histamine-2 receptor antagonist (H2RA) users; (iv) they included participants without chronic kidney disease (CKD) at baseline; and (v) they were either cohort studies or randomized controlled trials. Studies were excluded if they were literature or systematic reviews, letters to the editor, animal studies, or involved participants under the age of 18 years.

### 2.3. Study Outcome

The primary endpoint is the incidence of CKD. The definition of incident CKD in each study is provided in Table 1.

### 2.4. Data Extraction and Quality Assessment of Included Studies

The data from the study were independently extracted by two authors (JI and JEC). Any discrepancies identified were cross-verified with the original article and resolved through the consensus of a third author (SPA). Two authors (SPA and JEC) meticulously extracted data from eligible studies, capturing critical details such as demographics, study designs, follow-up durations, and clinical outcomes for both patient groups into a structured spreadsheet. The methodological integrity of each study was appraised by two authors (SPA and JEC) employing the Newcastle–Ottawa Scale for observational studies [23]. Any methodological disputes were adjudicated by a third author (JI). To assess the certainty of evidence, the Grading of Recommendations Assessment, Development, and Evaluation (GRADE) approach was used [24].

### 2.5. Statistical Analysis

We performed a conventional meta-analysis for primary outcomes using the random-effect model and the DerSimonian-and-Laird method to account for the study variations. Initially, we assessed the outcome by comparing proton pump inhibitor (PPI) users with non-PPI users. Subsequently, we performed a secondary comparison, evaluating the outcome between individuals using PPIs and those taking H2RAs. Where possible, we selected the most adjusted effect sizes or those derived from propensity-score matching or weighting for inclusion in the pooled analysis. The covariates used in these adjustments are detailed and presented in Table 1. The results were expressed as pooled hazard ratios (HRs) with their corresponding 95% confidence intervals (95% CIs). To study the variability among studies, we employed the Higgins I-square (I^2^) test [25]. Here, I^2^ values below 75% were deemed to represent mild to moderate heterogeneity, while values above 75% indicated significant heterogeneity. To explore the reason for high heterogeneity, we conducted a sensitivity analysis utilizing a leave-one-out approach and subgroup analyses based on study design (prospective vs. retrospective) and follow-up period (≤5 years vs. >5 years). Furthermore, the detection of publication bias was conducted for outcomes using funnel plots and Egger’s regression test [26]. All statistical analyses and the generation of graphical representations were conducted using STATA software, version 17.0 (Stata Corp. LLC, College Station, TX, USA).

## 3. Results

### 3.1. Baseline Characteristics of Studies

Our analysis included 11 studies with a total of 1,144,056 participants (310,331 on PPIs, 793,936 not on PPIs, and 39,789 on H2RAs), with follow-up periods ranging from three months to 14 years. Among these, five studies adopted a prospective design [15,18,20], while six were retrospective [12,13,16,19,21,27]. Notably, the study by Moayyedi et al. [22] was a double-blind, placebo-controlled randomized controlled trial, The Cardiovascular Outcomes for People Using Anticoagulation Strategies (COMPASS) trial (ClinicalTrials.gov Identifiers: NCT01776424). Furthermore, a study by Kweon et al. investigated the use of PPIs using the National Health Insurance Service–National Sample Cohort (NHIS-NSC) and a multicenter electronic health record comprising six hospitals in Korea [13]. In addition, Lazarus et al. conducted a population-based study using data from the Atherosclerosis Risk in Communities (ARIC) study and separately analyzed a validation cohort with data from the Geisinger Health System (GHS) [15].

### 3.2. Risk of Bias Assessment of Included Studies

The process of study screening and selection is comprehensively delineated in Supplementary Figure S1. We assessed the methodological quality and risk of bias of the included studies using the Newcastle–Ottawa Scale (NOS). The studies we analyzed achieved NOS scores between 7 and 9 stars, suggesting their high methodological quality. These scores reflect the robustness of the study designs, the adequacy of selection processes, the comparability of groups, and the assessment of outcomes. Using the GRADE approach, the certainty of the evidence was assessed as “very low”, largely because of the nature of the included studies which is mainly observational. Details regarding the quality evaluation of each study and the certainty of evidence are available in Supplementary Tables S2, S3 and S4, respectively.

### 3.3. Baseline Characteristics of Patients

In this study, patients were divided into two cohorts to enable a comparative analysis of treatment outcomes. The initial cohort analysis compared patients receiving PPIs (n = 101,244) with those not receiving PPIs (n = 373,848). At baseline, the PPI cohort was younger on average but exhibited a higher prevalence of comorbid conditions such as hypertension and diabetes mellitus. Additionally, the PPI cohort contained fewer smokers compared to the non-PPI group. The subsequent cohort analysis assessed outcomes between patients prescribed PPIs (n = 202,466) and those receiving histamine-2 receptor antagonists (H2RAs) (n = 39,789). Patients on PPIs were typically older and more frequently male compared to those on H2RAs. This group also showed a greater prevalence of hypertension, diabetes mellitus, and hyperlipidemia than the H2RA cohort. Details of baseline characteristics are presented in Table 2.

### 3.4. Meta-Analysis of Outcomes

**Figure 1:**
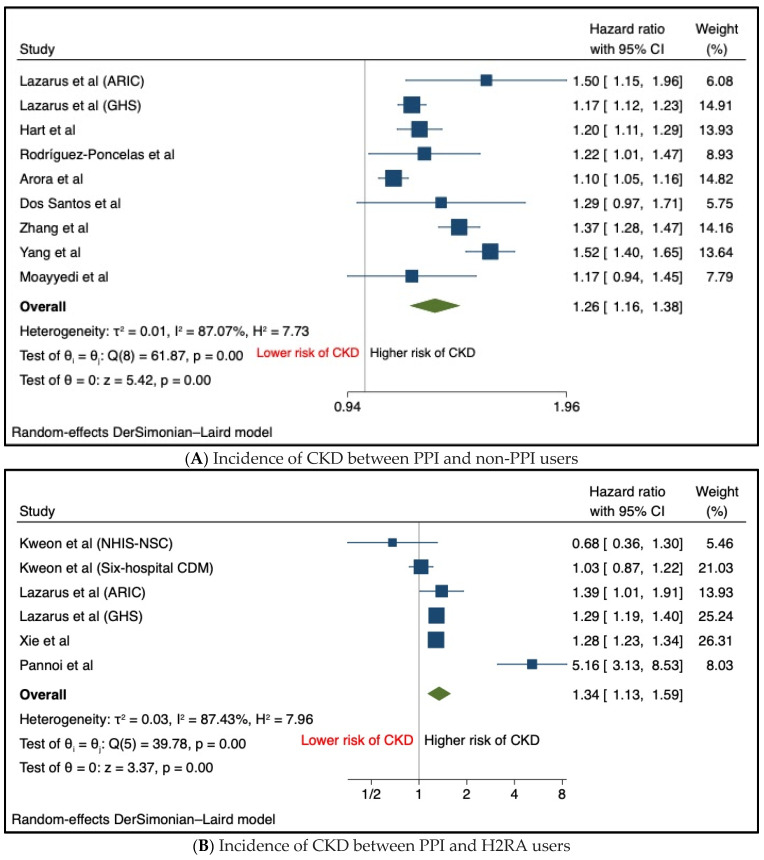
Forest plot of primary outcome—incidence of CKD; (**A**) between PPI and non-PPI users and (**B**) between PPI and H2RA users [10,12,13,14,15,16,18,20,21,22,27].

Eight studies were evaluated to compare the incidence of chronic kidney disease (CKD) between patients with and without the use of PPIs. The meta-analysis results demonstrated a significant association between PPI use and an elevated risk of CKD (HR: 1.26, 95% CI: 1.16–1.38, *p* < 0.001) (Figure 1A), indicative of an increased risk when compared to non-PPI users.

Additionally, four studies encompassing six patient cohorts compared the incidence of CKD in individuals using PPIs against those using H2RAs. The findings indicate that CKD risk was notably higher among PPI users in comparison to H2RA users (HR: 1.34, 95%, CI: 1.13–1.59, *p* < 0.001) (Figure 1B), suggesting a statistically significant increased risk of CKD associated with PPI usage relative to H2RAs.

### 3.5. Sensitivity Analysis and Subgroup Analysis

In the sensitivity analyses, we used leave-one-out techniques to evaluate each outcome, observing that the results retained a similar magnitude and direction across both assessed outcomes following this method, suggesting the robustness of the primary analysis. Additionally, we conducted subgroup analyses stratified by duration of follow-up (≤5 years versus >5 years) and study design (prospective versus retrospective). These analyses revealed no significant differences between subgroups based on follow-up period and study design when comparing PPIs with non-PPIs and H2Ras, respectively (Supplementary Figures S2–S5).

### 3.6. Publication Bias

Publication bias was evaluated through the visualization of funnel plots and the application of Egger’s regression test. The funnel plots appeared nearly symmetrical for both outcomes, and the results of Egger’s regression test were not significant (*p* > 0.05), indicating no evidence of publication bias (Supplementary Figures S6 and S7).

## 4. Discussion

We compiled 11 studies with over 1 million patients, of which 27% of patients commenced the use of PPIs. Our results suggested that PPI use is associated with an increased risk of CKD compared to non-PPI users and H2RA users, respectively. These results remained consistent after a sensitivity analysis and subgroup analyses based on follow-up period and study design.

The association between PPIs and the risk of acute interstitial nephritis (AIN) or acute kidney injury (AKI) has been previously described. Blank and colleagues examined over 500,000 patients who started to use PPIs in New Zealand and found that current use of PPIs was associated with an increased risk of AIN with a crude incidence rate of 11.98 per 100,000 person-years [15,29]. Antoniou et al. conducted a population-based study in Ontario and discovered that those who started PPI therapy had a higher rate of AKI (HR: 2.52, 95% CI: 2.27 to 2.79) and AIN (HR: 3.00, 95%, CI: 1.47 to 6.14) compared to controls [30]. The onset of PPI-induced interstitial nephritis typically manifests as an unpredictable, idiosyncratic hypersensitivity reaction to the drug or its by-products. Notably, this type of adverse effect is independent of both dosage and duration of exposure and thus presents substantial challenges in identifying individuals at risk prior to clinical manifestations [31].

In the majority of cases, it has been proposed that CKD in the setting of PPIs develops in the setting of AIN and AKI which does not result in complete recovery, resulting in an AKI to CKD continuum. However, there is evidence that PPI-associated CKD may occur without antecedent AKI [32]. Hypothetically PPIs may cause AKI and CKD via a variety of mechanisms. There has been mounting evidence implicating reactive oxygen species-mediated injury, changes in liposomal pH, lipid peroxidation, endothelial dysfunction, endothelial senescence, increases in intracellular calcium mediated tubular necrosis, immunologic injury, and mitochondrial dysfunction [33,34]. In general, AIN is the most common type of AKI associated with PPI therapy [34,35,36]. Although the exact mechanism of PPI associated with AIN has not been completely elucidated, evidence suggests that immunologic processes are involved. Recent evidence supports the involvement of Interleukin-17 (IL-17) and Th-1-mediated tubulitis [37]. Berney-Meyer et al. evaluated 25 renal biopsy specimens of Omeprazole-associated AIN which revealed a predominant mononuclear cell infiltrate and tubulitis in most cases. There were no significant eosinophilic infiltrates, and glomeruli were not involved. Immunostaining revealed that in the majority of cases, CD 4+ lymphocytes were the predominant cell type. In addition, co-staining of CD4, IL-17A, and IL-17F occurred in 44–48% of all cases, supporting a Th-17-mediated inflammatory process. T-bet+ cell infiltrates were also present, indicating additional Th-1 involvement [37]. In addition, PPIs have been known to cause hypomagnesemia, which is associated with endothelial dysfunction, oxidative stress, mitochondrial dysfunction, decreased nitric oxide production, and the increased synthesis of pro-inflammatory cytokines, IL-6, and TNF-α [38]. Thus, PPI-induced hypomagnesemia may perpetuate AIN and reactive oxygen species oxidative stress-mediated acute tubular necrosis. PPIs are known to cause hypocalcemia, which results in increases in intracellular calcium. Elevations in intracellular calcium result in a p38MAPK-mediated inflammatory response [34]. PPIs also induce an increase in caspase-3 activity and endothelin-1 production, resulting in renal vasoconstriction and ensuing acute tubular injury [34,39].

Renal tubular injury and AIN associated with PPI therapy are initially mild, asymptomatic, and often subclinical, usually taking weeks or months to become clinically recognized [40]. Furthermore, PPI-associated AIN may lack the usual signs and symptoms of a hypersensitivity reaction such as fever and rash [11,40]. The under-recognition of mild renal tubular injury resulting in the failure of withdrawing PPIs may lead to a recurrent cycle of AIN, ATN, and CKD. PPI-induced hypomagnesemia may lead to progressive endothelial cell dysfunction, pro-inflammatory cytokine-associated chronic inflammation, and the development of tubulointerstitial fibrosis. Alves et al. and others demonstrated that hypomagnesemia was an independent risk factor for non-recovery of AKI in critically ill patients, possibly leading to limited recovery from AKI [41]. Additionally, hypomagnesemia may perpetuate further CKD by increasing the tubular load of phosphate [28,34,42].

While our study found a significant association between PPI and the development of CKD, the pathophysiology underlying this remained unclear. Notably, it is documented that a sizable proportion of patients failed to achieve complete recovery of renal function following AIN [17]. This lack of recuperation could be attributed to the rapid progression to interstitial fibrosis following the acute inflammatory response. Consequently, this persistent impairment in renal function, potentially coupled with ongoing chronic interstitial nephritis, constitutes a precursor for CKD [17]. Moreover, given the correlation between PPI use and AKI, individuals experiencing recurrent AKI episodes may exhibit an increased likelihood of progressing to chronic kidney disease (CKD) [43].

## 5. Strengths and Limitations

This study represents one of the first meta-analyses to explore the association between proton pump inhibitor (PPI) use and the incidence of chronic kidney disease (CKD). Its strengths include the comprehensive nature of the analysis, the large sample size, and the robustness of the analyses. Moreover, our analysis incorporated data from diverse geographic regions, enabling us to assess potential population-specific effects and thereby enhance the generalizability of our findings.

The principal limitation of this study is that it predominantly incorporates observational studies, which are inherently prone to confounding bias. In addition, the heterogeneity for the outcomes is substantial and should be taken into consideration upon results interpretation. We conducted sensitivity analyses to explore the source of heterogeneity and found that results remained consistent after the exclusion of one study at a time.

In addition, to address concerns regarding the low statistical power of the funnel plots presented in Supplementary Figures S8 and S9, we employed Egger’s regression test as an additional method to assess potential publication bias. Furthermore, patients on PPIs exhibited a greater comorbidity burden, including a higher prevalence of hypertension and diabetes, compared to non-users and those using H2 receptor antagonists (H2RAs). It is important to note that the majority of the studies included in our analysis accounted for these factors of poly-pathology along with baseline demographics such as gender and age, either through multivariate logistic regression or by adjustments made after propensity score weighting. The specific covariates or confounders adjusted for are detailed in the Supplementary Table S4.

Importantly, granular data including the presence of proteinuria, changes in estimated glomerular filtration rate (eGFR) or creatinine, or a combination of these parameters were not consistently reported across the included studies. Additionally, the indications for PPI use were not extensively examined in the current literature due to a lack of reported data. However, it should be noted that the authors of each included study made significant efforts to adjust for potential confounders, including comorbidities, peptic ulcer disease, and concurrent medications. Lastly, we were unable to analyze the relationship between individual types of PPIs and the risk of CKD, owing to the insufficient number of studies providing this detailed breakdown. Future research should aim to address these gaps by focusing on the detailed collection and analysis of these variables to enhance our understanding of the relationship between PPI use and CKD risk.

Moreover, the current literature employed different control groups when comparing PPI use and the risk of CKD. Numerous studies have examined the association between H2Ras and the risk of CKD, finding no increased risk associated with H2RA use. Our study employed various methods to assess CKD risk among PPI users. Initially, we compared PPI users with non-users; subsequently, we contrasted PPI users with an active control group, namely those using H2RAs. This comparative approach enabled us to more accurately evaluate the specific effects of PPIs on CKD risk relative to H2RAs. Our findings indicate a statistically significant increase in CKD risk in PPI users compared to both non-users and H2RA users, suggesting the potential renal implications of PPIs. Future research should continue to investigate the impact of different types and doses of H2RAs on CKD risk to fully elucidate their safety profile in the context of renal health. This detailed analysis is crucial for determining whether the increased risk of CKD is specific to PPI use or a broader consequence of acid suppression therapy.

## 6. Conclusions

Our multifaceted meta-analysis revealed that the use of PPIs is associated with a higher incidence of CKD compared to both non-PPI use and the use of H2RAs. These findings underscore the need for increased vigilance and prudent management of long-term PPI therapy. The widespread, often inappropriate use of PPIs, which frequently extends beyond recommended treatment durations, should be curtailed to mitigate the risk of adverse renal outcomes. It is imperative that healthcare providers reassess the necessity of PPI therapy regularly. Further extensive, prospective longitudinal studies are essential to confirm these findings and to explore mechanisms underlying the association between PPI use and increased CKD risk, which may inform future guidelines and interventions.

## Figures and Tables

**Table 1:** Characteristics of the included studies.

Study	Study Period	Study Design	Control Group	Type of PPI	Definition of CKD
Kweon et al. (NHIS-NSC) [13]	2002–2013	Retrospective	H2RAs	dexlansoprazole, esomeprazole, lansoprazole, omeprazole, pantoprazole, and rabeprazole	ICD-10 codes
Kweon et al. (6-hospital CDM) [13]	1999–2018	Retrospective	H2RAs	dexlansoprazole, esomeprazole, lansoprazole, omeprazole, pantoprazole, and rabeprazole	ICD-10 codes
Lazarus et al. (ARIC) [15]	1987–2011	Prospective	Non-PPIs, H2RAs	N/A	ICD-9 codes
Lazarus et al. (GHS) [15]	1997–2014	Prospective	Non-PPIs, H2RAs	N/A	eGFR < 60 mL/min/1.73 m^2^, or the development of ESRD
Hart et al. [16]	1993–2008	Retrospective	Non-PPIs	esomeprazole, lansoprazole, omeprazole, pantoprazole, or rabeprazole	ICD-9-CM code or an eGFR of less than 60 mL/min/1.73 m^2^
Rodríguez-Poncelas et al. 23]	2005–2012	Retrospective	Non-PPIs	omeprazole, esomeprazole, pantoprazole, lansoprazole, and rabeprazole	eGFR< 60 mL/min/1.73 m^2^ and/or UACR ≥ 30 mg/g, in two or more determinations in a period of a minimum of 3 months
Arora et al. [12]	2001–2008	Retrospective	Non-PPIs	N/A	observed eGFR < 60 mL/ min/1.73 m^2^
Xie et al. [17]	2006–2008		H2RAs	esomeprazole, lansoprazole, omeprazole, pantoprazole, or rabeprazole	2 eGFRs < 60 mL/min per 1.73 m^2^ at least 90 days apart
Dos Santos et al. [18]	2008–2014	Prospective	Non-PPIs	omeprazole, esomeprazole, lansoprazole, pantoprazole, and rabeprazole	2 eGFRs < 60 mL/min per 1.73 m^2^ at least 90 days apart
Pannoi et al. [19]	2010–2012	Retrospective	H2RAs	omeprazole, pantoprazole, dexlansoprazole, lanzoprazole, esomeprazole, and rabeprazole	ICD-10 codes
Zhang et al. [20]	2006–2010	Prospective	Non-PPIs	omeprazole, lansoprazole, pantoprazole, rabeprazole, and esomeprazole.	CKD variables provided by UK BioBank
Yang et al. [21]	2002–2013	Retrospective	Non-PPIs	esomeprazole, lansoprazole, omeprazole, pantoprazole, and rabeprazole	ICD-9 codes
Moayyedi et al. [22]	2013–2016	Randomized controlled trial	Non-PPIs	pantoprazole	N/A

CKD: chronic kidney disease; ESRD: end-stage renal disease; GFR: glomerular filtration rate; H2Ras: histamine-2 receptor antagonists; ICD: International Classification of Diseases; N/A: not available; PPI: proton pump inhibitors; UACR: urine albumin creatinine ratio.

**Table 2:** Characteristics of patients using proton pump inhibitors (PPIs), not using PPIs, and using histamine-2 receptor antagonists (H2RAs).

Study	Number of Patients, n			Age			Female			Hypertension			DM			CVD			Smoking						Peptic Ulcer Disease		
	PPI	Non-PPI	H2RA	PPI	Non-PPI	H2RA	PPI	Non-PPI %	H2RA %	PPI %	Non-PPI %	H2RA %	PPI %	Non-PPI %	H2RA %	PPI %	Non-PPI %	H2RA %	PPI %	Non-PPI %	H2RA %	PPI %	Non-PPI %	H2RA %	PPI	Non-PPI	H2RA
Kweon et al. (NHIS-NSC) [13]	1869	N/A	1869	N/A	N/A	N/A	51.5	N/A	51.4	51.0	N/A	51.3	21.1	N/A	21.3	16.1	N/A	17.7	N/A	N/A	N/A	57.6	N/A	57.3	N/A	N/A	N/A
Kweon et al. (6-hospital CDM) [13]	5967	N/A	5967	N/A	N/A	N/A	53.7	N/A	55.5	26.9	N/A	27.4	9.8	N/A	9.5	16.1	N/A	16.8	N/A	N/A	N/A	56.0	N/A	54.6	N/A	N/A	N/A
Lazarus et al. (ARIC) [15]	322	9204	956	62.8 (5.5)	63.1 (5.5)	62.5 (5.6)	57.5	44.4	60.7	54.3	44.8	50.0	14.9	15.6	18.0	13.7	10.8	14.1	11.5	15.2	15.5	27.6	33.2	32.8	N/A	N/A	N/A
Lazarus et al. (GHS) [15]	16,900	225,211	6640	50.0 (15.9)	50.3 (16.3)	49.5 (16.3)	56.8	56.5	N/A	33.3	30.2	34.0	10.8	10.4	9.7	11.3	8.7	11.8	25.7	23.9	26.1	13.9	9.5	14.4	N/A	N/A	N/A
Hart et al. [16]	12,093	12,093	N/A	51.4 (17.2)	50.9 (16.8)	N/A	61.7	61.2	N/A	24.9	26.6	N/A	9.6	9.2	N/A	N/A	N/A	N/A	N/A	N/A	N/A	33.3	34.4	N/A	N/A	N/A	N/A
Rodríguez-Poncelas et al. [27]	5254	382	N/A	N/A	N/A	N/A	N/A	N/A	N/A	N/A	N/A	N/A	N/A	N/A	N/A	N/A	N/A	N/A	N/A	N/A	N/A	N/A	N/A	N/A	N/A	N/A	N/A
Arora et al. [12]	22,734	53,728	N/A	56.3 (13.17)	56.94 (15.38)	N/A	5.9	6.2	N/A	62.5	62.3	N/A	17.5	21.2	N/A	N/A	N/A	N/A	N/A	N/A	N/A	N/A	N/A	N/A	N/A	N/A	N/A
Xie et al. [17]	173,321	N/A	20,270	56.85 (11.85)	N/A	55.40 (12.81)	7	N/A	6.6	78.9	N/A	78.0	41.7	N/A	44.0	41.4	N/A	41.7	N/A	N/A	N/A	N/A	N/A	N/A	15.1	N/A	3.3
Dos Santos et al. [18]	1005	12,296	N/A	54.4 (9.0)	51.1 (8.7)	N/A	57.4	54.7	N/A	45.0	32.5	N/A	11.1	8.7	N/A	0.0	5.8	N/A	10.1	13.0	N/A	3.4	1.6	N/A	N/A	N/A	N/A
Pannoi et al. [19]	4087	N/A	4087	N/A	N/A	N/A	69	N/A	66.2	2.8	N/A	1.7	0.7	N/A	0.3	0.0	N/A	0.4	N/A	N/A	N/A	23.2	N/A	23.6	N/A	N/A	N/A
Zhang et al. [28]	28,151	28,151	N/A	58.89 (7.49)	58.89 (7.48)	N/A	54.7	54.7	N/A	N/A	N/A	N/A	10.7	10.7	N/A	16.2	16.2	N/A	11.5	11.5	N/A	18.3	18.3	N/A	7.0	7.0	N/A
Yang et al. [21]	5994	23,976	N/A	59.1 (11.9)	59.1 (11.9)	N/A	40.5	40.5	N/A	35.5	35.6	N/A	N/A	N/A	N/A	9.5	9.2	N/A	N/A	N/A	N/A	93.2	84.0	N/A	N/A	N/A	N/A
Moayyedi et al. [22]	8791	8807	N/A	67.6 (8.1)	67.7 (8.1)	N/A	22	21.2	N/A	N/A	N/A	N/A	38.3	38.3	N/A	61.5	61.4	N/A	23.5	22.8	N/A	4.8	5.1	N/A	2.6	2.5	N/A

Categorical variables are expressed as n (%). Continuous variables are expressed as the mean (standard deviation). CVD: cardiovascular diseases; DM: diabetes mellitus; H2RA: histamine-2 receptor antagonists; N/A: not available; PPI: proton pump inhibitors.

## Data Availability

All the data are available within the article and its Supplementary Materials.

## Supplementary Materials

The following supporting information can be downloaded at: https://www.mdpi.com/article/10.3390/biomedicines12071414/s1.

## Abbreviations

CKD	chronic kidney disease
H2RA	histamine-2 receptor antagonists
PPI	proton pump inhibitors
