# Supplementary material for: Association between Proton Pump Inhibitor Use and Risk of Incident Chronic Kidney Disease: Systematic Review and Meta-Analysis
Source: Biomedicines. 2024 Jun 25;12(7):1414. doi: 10.3390/biomedicines12071414 (PMC11274577; doi:10.3390/biomedicines12071414)
Supplement: Supplementary file 1 [file biomedicines-12-01414-s001.zip › biomedicines-3023998-supplementary.pdf]

Supplementary Table S1 PRISMA 2020 Checklist
Supplementary Table S2 Risk of bias assessment using Newcastle-Ottawa-Scale for observational studies
Supplementary Table S3 Certainty of evidence using GRADE approach
Supplementary Table S4 Covariates adjustment in the included studies
Supplementary Figure S1 PRISMA Flow Diagram
Supplementary Figure S2 Sensitivity analysis for incidence of CKD between PPIs and non-PPIs group using leave-one-out approach
Supplementary Figure S3 Sensitivity analysis for incidence of CKD between PPIs and H2RAs group using leave-one-out approach
Supplementary Figure S4 Subgroup analysis for incidence of CKD between PPIs and non-PPIs group based on follow-up period
Supplementary Figure S5 Subgroup analysis for incidence of CKD between PPIs and H2RAs group based on follow-up period
Supplementary Figure S6 Subgroup analysis for incidence of CKD between PPIs and non-PPIs group based on study design
Supplementary Figure S7 Subgroup analysis for incidence of CKD between PPIs and H2RAs group based on study design
Supplementary Figure S8 Funnel plot for incidence of CKD between PPIs and non-PPIs group
Supplementary Figure S9 Funnel plot for incidence of CKD between PPIs and H2RAs group

Supplementary Table S1 PRISMA 2020 Checklist

Section and Topic	Item #	Checklist item	Location where item is reported
**TITLE**			
**Title**	**1**	**Identify the report as a systematic review.**	**Page 1, 2**
**ABSTRACT**			
**Abstract**	**2**	**See the PRISMA 2020 for Abstracts checklist.**	**-**
**INTRODUCTION**			
**Rationale**	**3**	**Describe the rationale for the review in the context of existing knowledge.**	**Page 1, 2**
**Objectives**	**4**	**Provide an explicit statement of the objective(s) or question(s) the review addresses.**	**Page 2**
**METHODS**			
**Eligibility criteria**	**5**	**Specify the inclusion and exclusion criteria for the review and how studies were grouped for the syntheses.**	**Page 2**
**Information sources**	**6**	**Specify all databases, registers, websites, organisations, reference lists and other sources searched or consulted to identify studies. Specify the date when each source was last searched or consulted.**	**Page 2**
**Search strategy**	**7**	**Present the full search strategies for all databases, registers and websites, including any filters and limits used.**	**Page 2, Supplementary Table 1**
**Selection process**	**8**	**Specify the methods used to decide whether a study met the inclusion criteria of the review, including how many reviewers screened each record and each report retrieved, whether they worked independently, and if applicable, details of automation tools used in the process.**	**Page 2**
**Data collection process**	**9**	**Specify the methods used to collect data from reports, including how many reviewers collected data from each report, whether they worked independently, any processes for obtaining or confirming data from study investigators, and if applicable, details of automation tools used in the process.**	**Page 3**
**Data items**	**10a**	**List and define all outcomes for which data were sought. Specify whether all results that were compatible with each outcome domain in each study were sought (e.g. for all measures, time points, analyses), and if not, the methods used to decide which results to collect.**	**Page 3**
	**10b**	**List and define all other variables for which data were sought (e.g. participant and intervention characteristics, funding sources). Describe any assumptions made about any missing or unclear information.**	**Page 3**
**Study risk of bias assessment**	**11**	**Specify the methods used to assess risk of bias in the included studies, including details of the tool(s) used, how many reviewers assessed each study and whether they worked independently, and if applicable, details of automation tools used in the process.**	**Page 3,4**

Effect measures	12	Specify for each outcome the effect measure(s) (e.g. risk ratio, mean difference) used in the synthesis or presentation of results.	Page 4
Synthesis methods	13a	Describe the processes used to decide which studies were eligible for each synthesis (e.g. tabulating the study intervention characteristics and comparing against the planned groups for each synthesis (item #5)).	Table 1
	13 b	Describe any methods required to prepare the data for presentation or synthesis, such as handling of missing summary statistics, or data conversions.	Page 4
	13c	Describe any methods used to tabulate or visually display results of individual studies and syntheses.	Page 4
	13 d	Describe any methods used to synthesize results and provide a rationale for the choice(s). If meta-analysis was performed, describe the model(s), method(s) to identify the presence and extent of statistical heterogeneity, and software package(s) used.	Page 4
	13e	Describe any methods used to explore possible causes of heterogeneity among study results (e.g. subgroup analysis, meta-regression).	Page 4
	13f	Describe any sensitivity analyses conducted to assess robustness of the synthesized results.	Page 4
Reporting bias assessmen t	14	Describe any methods used to assess risk of bias due to missing results in a synthesis (arising from reporting biases).	Page 3, 4
Certainty assessmen t	15	Describe any methods used to assess certainty (or confidence) in the body of evidence for an outcome.	Page 3, 4
RESULTS			
Study selection	16a	Describe the results of the search and selection process, from the number of records identified in the search to the number of studies included in the review, ideally using a flow diagram.	Page 4, Supplem entary Figure 1
	16 b	Cite studies that might appear to meet the inclusion criteria, but which were excluded, and explain why they were excluded.	Supplem entary Figure 1
Study characteris tics	17	Cite each included study and present its characteristics.	Page 4, Table 1
Risk of bias in studies	18	Present assessments of risk of bias for each included study.	Page 4, Supplem entary Table 2, 3

**Results of individual studies**	**19**	**For all outcomes, present, for each study: (a) summary statistics for each group (where appropriate) and (b) an effect estimate and its precision (e.g. confidence/credible interval), ideally using structured tables or plots.**	**Page 7**
**Results of syntheses**	**20a**	**For each synthesis, briefly summarise the characteristics and risk of bias among contributing studies.**	**Page 4, Supplementary Table 2, 3**
	**20b**	**Present results of all statistical syntheses conducted. If meta-analysis was done, present for each the summary estimate and its precision (e.g. confidence/credible interval) and measures of statistical heterogeneity. If comparing groups, describe the direction of the effect.**	**Page 7**
	**20c**	**Present results of all investigations of possible causes of heterogeneity among study results.**	**Page 7, 8**
	**20d**	**Present results of all sensitivity analyses conducted to assess the robustness of the synthesized results.**	**Page 7, 8**
**Reporting biases**	**21**	**Present assessments of risk of bias due to missing results (arising from reporting biases) for each synthesis assessed.**	**Page 4**
**Certainty of evidence**	**22**	**Present assessments of certainty (or confidence) in the body of evidence for each outcome assessed.**	**Page 4, Supplementary Table 3, 4**
**DISCUSSION**			
**Discussion**	**23a**	**Provide a general interpretation of the results in the context of other evidence.**	**Page 8**
	**23b**	**Discuss any limitations of the evidence included in the review.**	**Page 9, 10**
	**23c**	**Discuss any limitations of the review processes used.**	**Page 9, 10**
	**23d**	**Discuss implications of the results for practice, policy, and future research.**	**Page 9, 10**
**OTHER INFORMATION**			
**Registration and protocol**	**24a**	**Provide registration information for the review, including register name and registration number, or state that the review was not registered.**	**Page 2**
	**24b**	**Indicate where the review protocol can be accessed, or state that a protocol was not prepared.**	**Page 2**
	**24c**	**Describe and explain any amendments to information provided at registration or in the protocol.**	**Page 2**

**Support**	**25**	**Describe sources of financial or non-financial support for the review, and the role of the funders or sponsors in the review.**	**Page 10**
**Competing interests**	**26**	**Declare any competing interests of review authors.**	**Page 10**
**Availability of data, code and other materials**	**27**	**Report which of the following are publicly available and where they can be found: template data collection forms; data extracted from included studies; data used for all analyses; analytic code; any other materials used in the review.**	**Page 10**

Supplementary Table S2 Risk of bias assessment using Newcastle-Ottawa-Scale for observational studies for incidence of CKD between PPIs and non-PPIs group

**Study**	**Selection**				**Comparability**	**Exposure**			
	Representativeness of the exposed cohort	Selection of the non exposed cohort	Ascertainment of exposure	Demonstration that outcome of interest was not present at start of study	Comparability of cohorts on the basis of the design or analysis	Assessment of outcome	Was followed-up long enough for outcomes to occur	Adequacy of follow up of cohorts	Total score
Lazarus et al (ARIC)	★	★	★	★	★★	★	★	★	9
Lazarus et al (GHS)	★	★	★	★	★★	★	★	★	9
Hart et al	★	★	★	★	★★	★	★	0	8
Rodríguez-Poncelas et al	★	★	★	★	★★	★	★	0	8
Arora et al	★	★	★	★	★★	★	0	★	8
Dos Santos et al	★	★	★	★	★★	★	★	★	9
Zhang et al	★	★	★	★	★★	★	★	★	9
Yang et al	★	★	★	★	★★	★	0	0	7
Moayyedi et al	★	★	★	★	★★	0	★	★	8

Supplementary Table 2 Risk of bias assessment using Newcastle-Ottawa-Scale for observational studies for incidence of CKD between PPIs and H2RAs group

**Study**	**Selection**	**Comparability**	**Exposure**

	Representativeness of the exposed cohort	Selection of the non exposed cohort	Ascertainment of exposure	Demonstration that outcome of interest was not present at start of study	Comparability of cohorts on the basis of the design or analysis	Assessment of outcome	Was followed-up long enough for outcomes to occur	Adequacy of follow up of cohorts	Total score
Kweon et al (NHIS-CDM)	★	★	★	★	★★	★	★	★	9
Kweon et al (6-hospital-CDM)	★	★	★	★	★★	★	★	★	9
Lazarus et al (ARIC)	★	★	★	★	★★	★	★	★	9
Lazarus et al (GHS)	★	★	★	★	★★	★	★	★	9
Xie et al	★	★	★	★	★★	★	★	0	8
Pannoi et al	★	★	★	★	★★	★	★	★	9

Supplementary Table S3 Certainty of evidence using GRADE approach

**No of studies**	**Design**	**Risk of bias**	**Inconsistency**	**Indirectness**	**Imprecision**	**Other**	**Certainty (overall score)**
**Outcome: Incidence of CKD between PPIs and non-PPIs**							
8 studies with 9 cohorts	8 observational cohorts, 1 randomised study	not serious	serious	not serious	not serious	none	⊕⊖⊖⊖ very low
**Outcome: Incidence of CKD between PPIs and H2RAs**							
4 studies with 6 cohorts	Observational	not serious	serious	not serious	not serious	none	⊕⊖⊖⊖ very low

Supplementary Table S4 Covariates adjustment in the included studies

**Study**	**Effect size adjustment**
Kweon et al (NHIS-NSC)	PSM with gender, age group, index year, post-observation time, Charlson comorbidity index
Kweon et al (6-hospital CDM)	PSM with gender, age group, index year, post-observation time, Charlson comorbidity index
Lazarus et al (ARIC)	Age, sex, race, study center, education, health insurance status, baseline estimated glomerular filtration rate, urinary albumin creatinine ratio, cigarette smoking status, body mass index, systolic blood pressure, diabetes, cardiovascular disease, antihypertensive medication use, and anticoagulant medication use.
Lazarus et al (GHS)	Age, sex, race, eGFR, smoking status, BMI, systolic blood pressure, diabetes, history of cardiovascular disease, antihypertensive medication use, anticoagulant medication use, and statin, aspirin, and NSAID use.
Hart et al	PSM and further adjustment for heart failure, hypertension, hyperlipidemia, H. pylori infection, and use of H2-receptor blockers
Rodríguez-Poncelas et al	Age, gender, impaired fasting glucose, type 2 diabetes, obesity, high-normal blood pressure, hypertension, low-HDL-cholesterol, hightriglycerides level, metabolic syndrome, chronic diseases, tobacco consumption, alcohol consumption, cardiovascular disease, antihypertensive treatment, hypoglucemiant treatment, hypolipemiant treatment, non-steroidal antiinflammatory drugs, other countries origin.
Arora et al	Age, sex, race, GI and pre-PPI comorbidities
Xie et al	Baseline eGFR, age, race, sex, diabetes mellitus, hypertension, cardiovascular disease, peripheral artery disease, cerebrovascular disease, chronic lung disease, hepatitis C, HIV, dementia, gastroesophageal reflux disease, upper gastrointestinal tract bleeding, ulcer disease, H. pylori infection, Barrett esophagus, achalasia, stricture, and esophageal adenocarcinoma.
Dos Santos et al	Age, sex and per capita household income, excessive alcohol consumption, smoking, obesity, cardiovascular disease, diabetes, hypertension, use of NSAIDs, ARBs and ACEs.
Pannoi et al	Age category, sex, baseline eGFR(imputed), Steroids, Clopidogrel, and stratified, Charlson Comorbidity index (CCI), Hypertension, Steroid, and stratified by NSAID, entry year, and hospital visits assumingly different baseline hazard function between NSAID, entry year and hospital visit intervals at baseline

Zhang et al	Propensity-score weighted model with covariates including age, sex (male, female), ethnicity (white people or others), socioeconomic status (the index of multiple deprivation, fifth), smoking status (never smoker, previous smoker, or current smoker), alcohol consumption (daily or almost daily, one to four times a week, one to three times a month, and special occasions only or never), physical activity (low, moderate, or high), fruit and vegetable intake (≥5 portions or <5 portions), body mass index, systolic blood pressure, concomitant comorbidities (hyperlipidemia, diabetes, cardiovascular disease, gastroesophageal reflux disease, and peptic ulcer, yes or no), and medication use (including aspirin, non-aspirin NSAIDs, acetaminophen, antihypertensive drugs, statin, metformin, and H2RAs)
Yang et al	PSM and further adjustment for age, gender, hypertension, gout, CVA, IHD, PAD, CHF, socioeconomic status, urbanization and region
Moayyedi et al	No adjustment

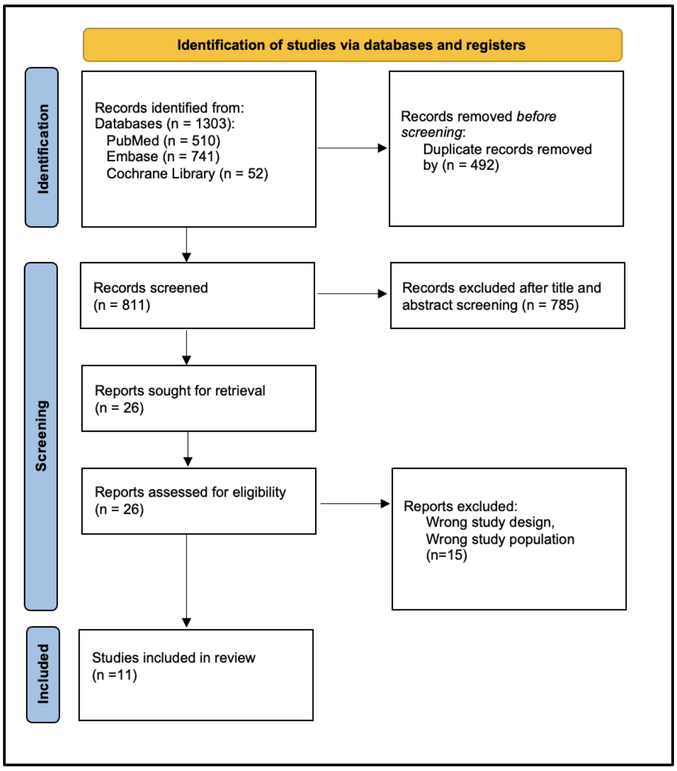

Supplementary Figure S1 PRISMA Flow Diagram

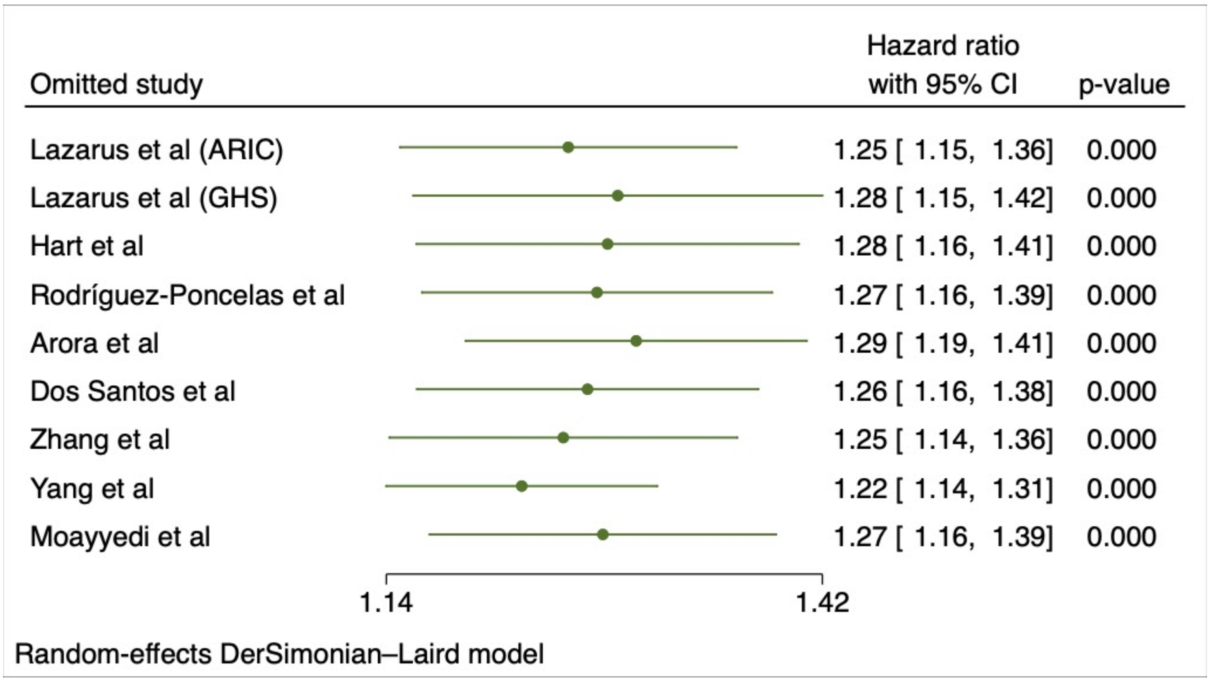

Supplementary Figure S2 Sensitivity analysis for incidence of CKD between PPIs and non-PPIs group using leave-one-out approach

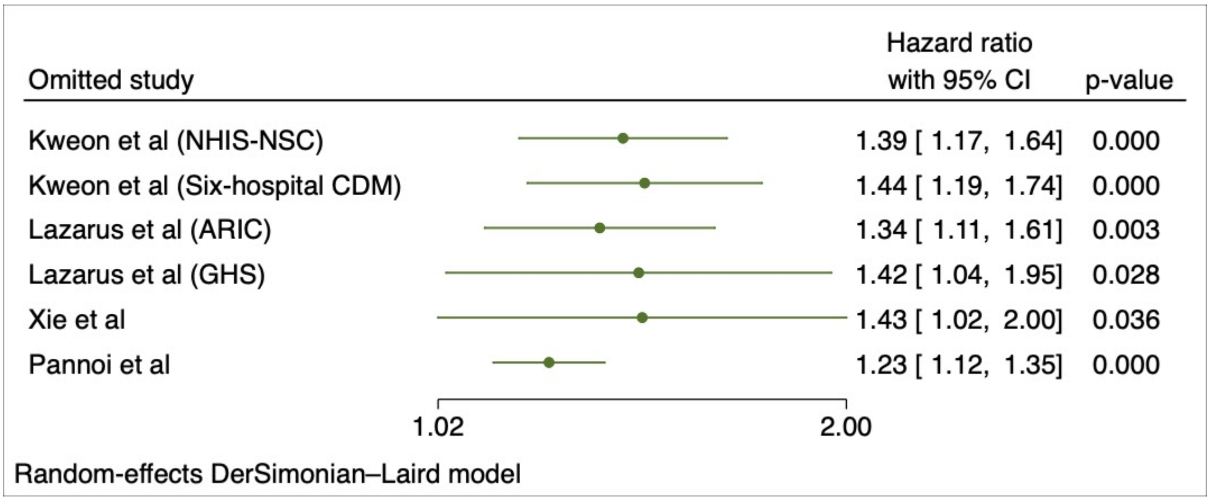

Supplementary Figure S3 Sensitivity analysis for incidence of CKD between PPIs and H2RAs group using leave-one-out approach

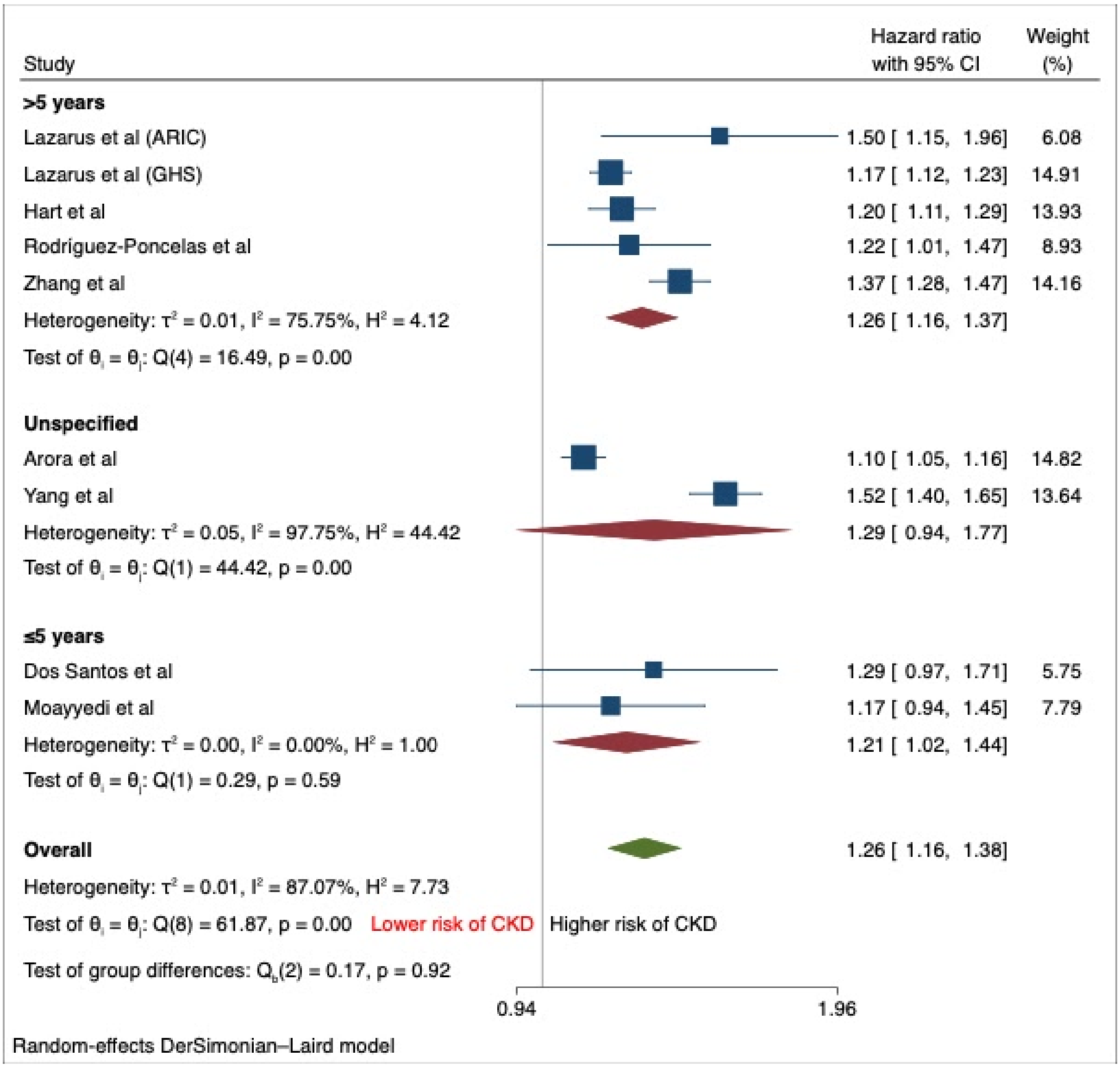

Supplementary Figure S4 Subgroup analysis for incidence of CKD between PPIs and non-PPIs group based on follow-up period

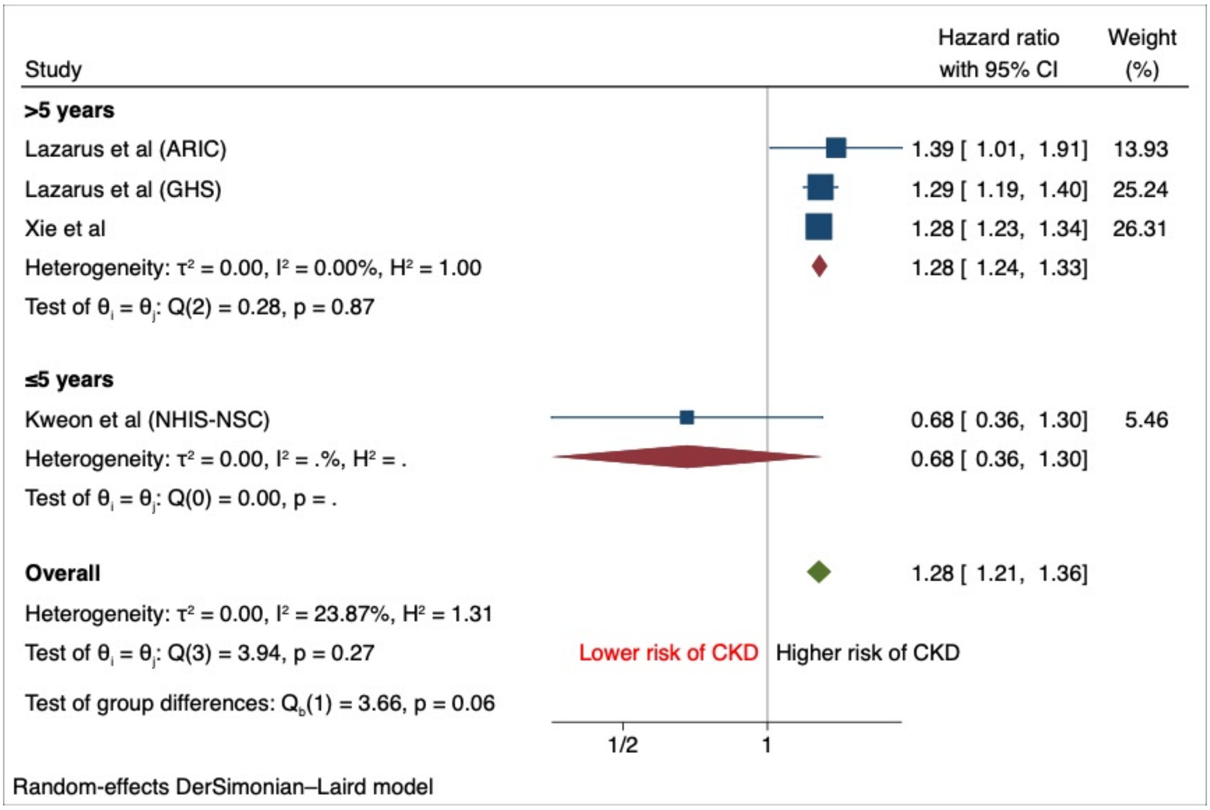

Supplementary Figure S5 Subgroup analysis for incidence of CKD between PPIs and H2RAs group based on follow-up period

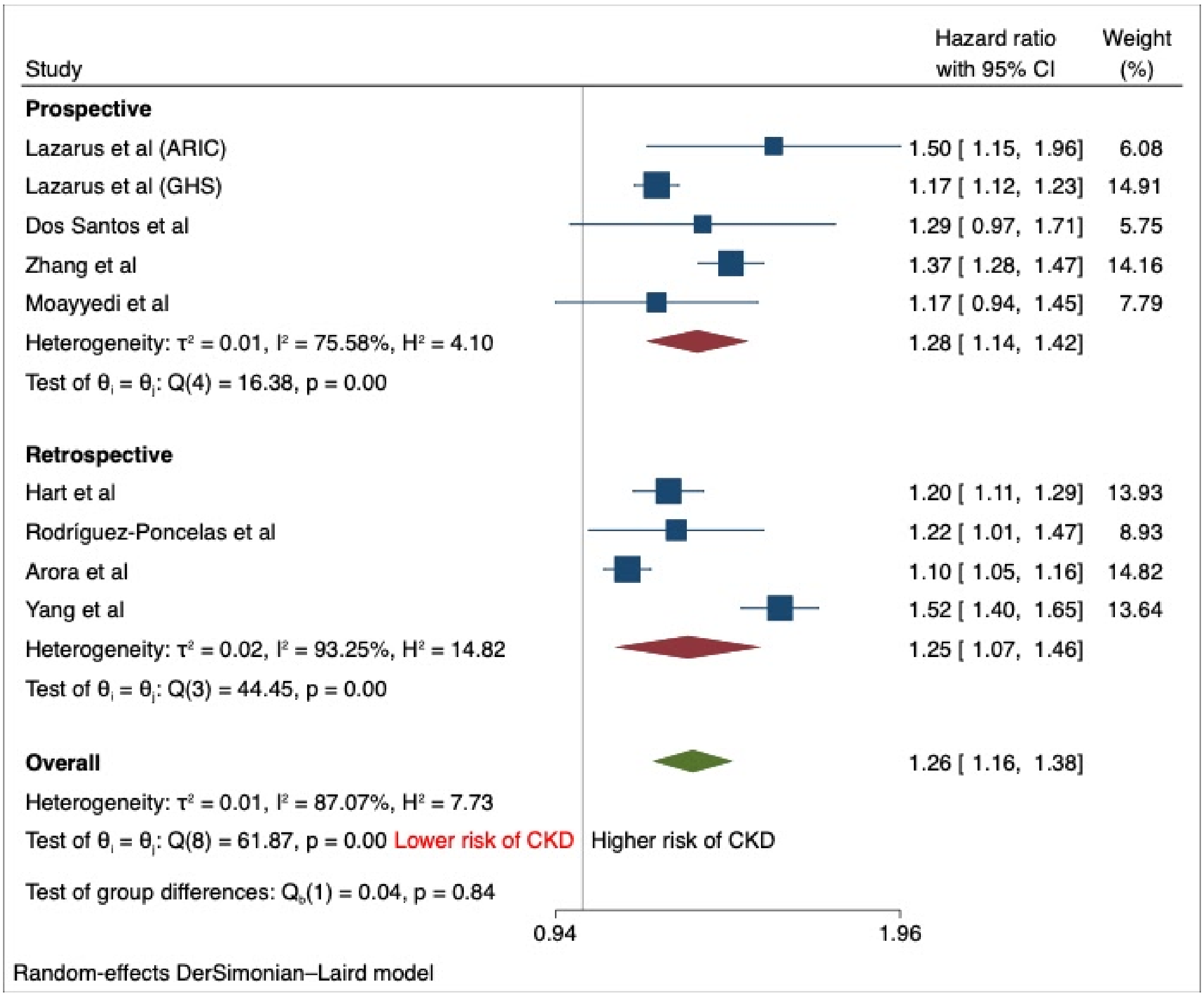

Supplementary Figure S6 Subgroup analysis for incidence of CKD between PPIs and non-PPIs group based on study design

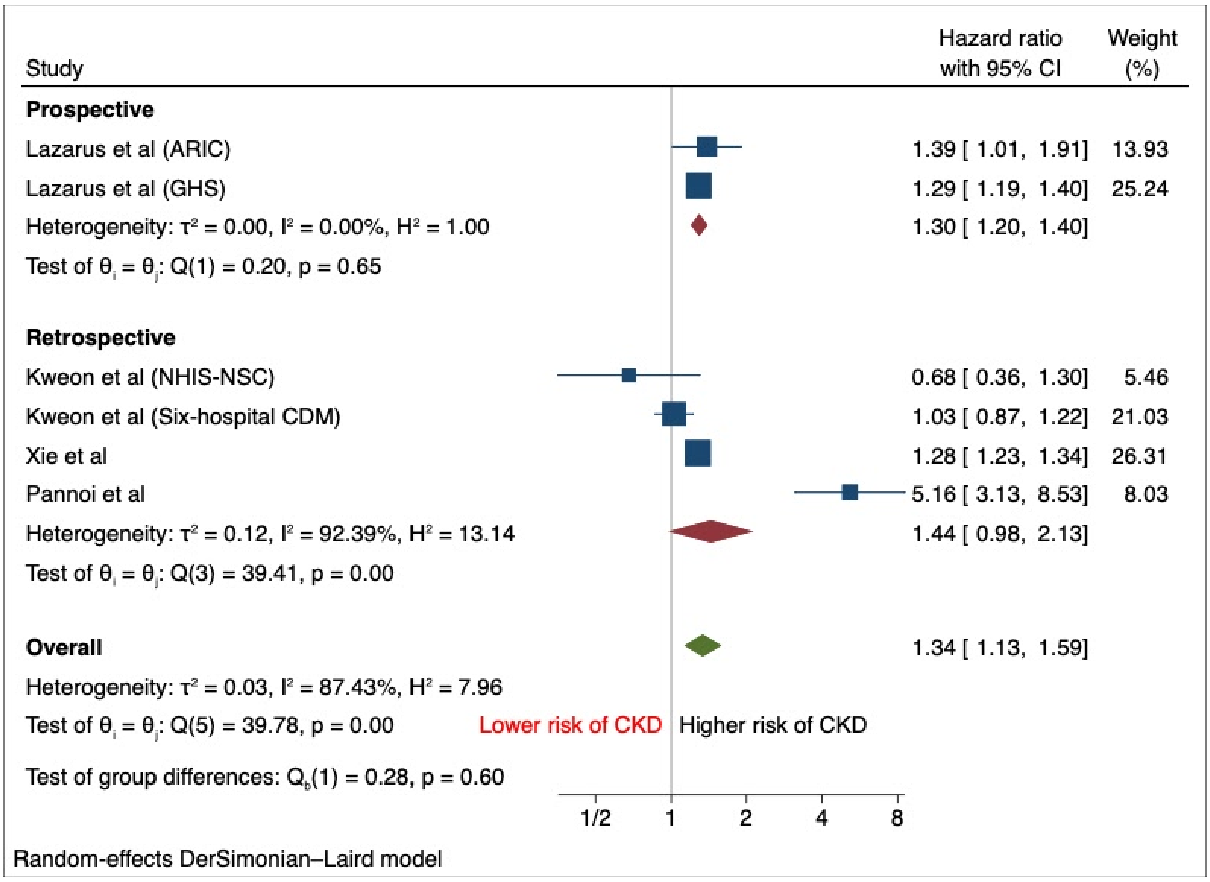

Supplementary Figure S7 Subgroup analysis for incidence of CKD between PPIs and H2RAs group based on study design

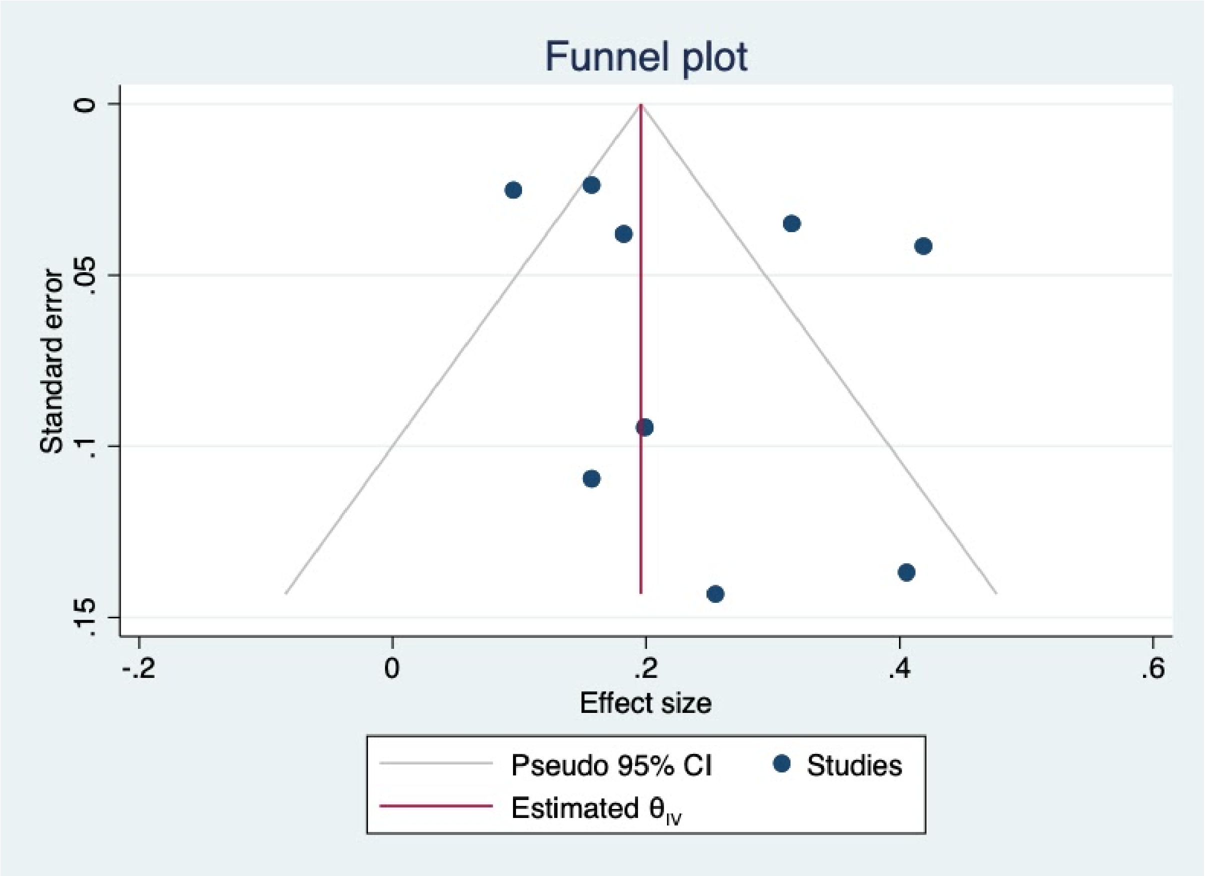

Supplementary Figure S8 Funnel plot for incidence of CKD between PPIs and non-PPIs group

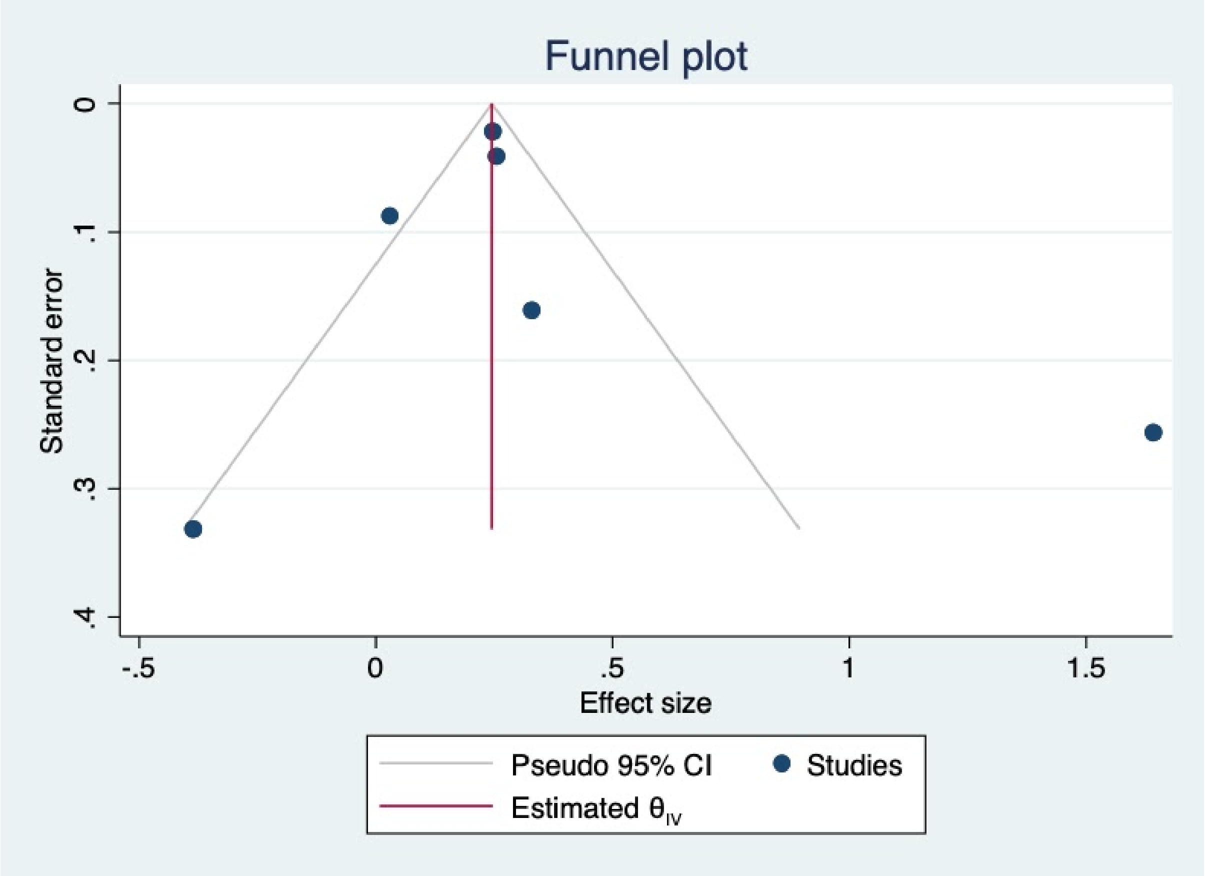

Supplementary Figure S9 Funnel plot for incidence of CKD between PPIs and H2RAs group
